# Modelling bluetongue and African horse sickness vector (*Culicoides* spp.) distribution in the Western Cape in South Africa using random forest machine learning

**DOI:** 10.1186/s13071-024-06446-8

**Published:** 2024-08-21

**Authors:** Joanna de Klerk, Michael Tildesley, Karien Labuschagne, Erin Gorsich

**Affiliations:** 1 The Zeeman Institute for Systems Biology and Infectious Disease Epidemiology Research, School of Life Sciences and Mathematics Institute, University of Warwick, Coventry, CV4 7AL UK; 2 Epidemiology, Parasites and Vectors, Agricultural Research Council, Onderstepoort Veterinary Research, Onderstepoort, 0110 South Africa

**Keywords:** Random forest, *Culicoides*, Ecological niche modelling, Vector-borne diseases

## Abstract

**Background:**

*Culicoides* biting midges exhibit a global spatial distribution and are the main vectors of several viruses of veterinary importance, including bluetongue (BT) and African horse sickness (AHS). Many environmental and anthropological factors contribute to their ability to live in a variety of habitats, which have the potential to change over the years as the climate changes. Therefore, as new habitats emerge, the risk for new introductions of these diseases of interest to occur increases. The aim of this study was to model distributions for two primary vectors for BT and AHS (*Culicoides imicola* and *Culicoides bolitinos*) using random forest (RF) machine learning and explore the relative importance of environmental and anthropological factors in a region of South Africa with frequent AHS and BT outbreaks.

**Methods:**

*Culicoides* capture data were collected between 1996 and 2022 across 171 different capture locations in the Western Cape. Predictor variables included climate-related variables (temperature, precipitation, humidity), environment-related variables (normalised difference vegetation index—NDVI, soil moisture) and farm-related variables (livestock densities). Random forest (RF) models were developed to explore the spatial distributions of *C. imicola, C. bolitinos* and a merged species map, where both competent vectors were combined. The maps were then compared to interpolation maps using the same capture data as well as historical locations of BT and AHS outbreaks.

**Results:**

Overall, the RF models performed well with 75.02%, 61.6% and 74.01% variance explained for *C. imicola, C. bolitinos* and merged species models respectively. Cattle density was the most important predictor for *C. imicola* and water vapour pressure the most important for *C. bolitinos*. Compared to interpolation maps, the RF models had higher predictive power throughout most of the year when species were modelled individually; however, when merged, the interpolation maps performed better in all seasons except winter. Finally, midge densities did not show any conclusive correlation with BT or AHS outbreaks.

**Conclusion:**

This study yielded novel insight into the spatial abundance and drivers of abundance of competent vectors of BT and AHS. It also provided valuable data to inform mathematical models exploring disease outbreaks so that *Culicoides*-transmitted diseases in South Africa can be further analysed.

**Graphical Abstract:**

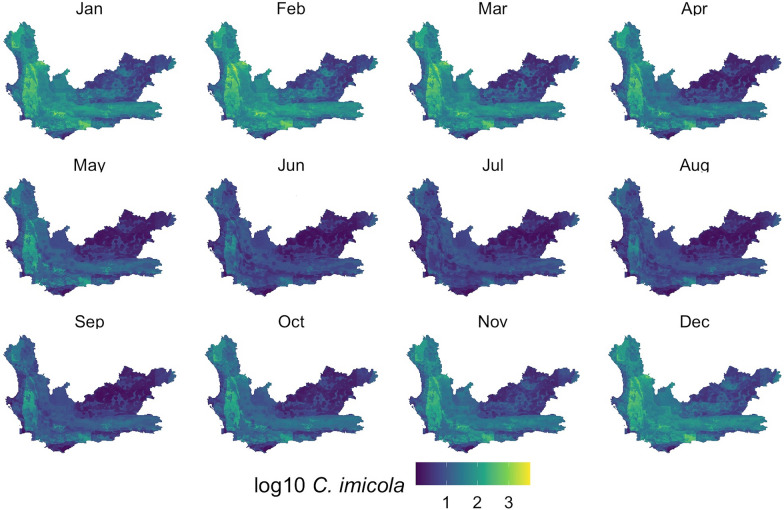

**Supplementary Information:**

The online version contains supplementary material available at 10.1186/s13071-024-06446-8.

## Background

*Culicoides* (Diptera: Ceratopogonidae) biting midges are blood-sucking insects responsible for the transmission of over 50 viruses, several of which are of significant global veterinary importance [1]. These include African horse sickness (AHS), bluetongue virus (BT), Schmallenberg virus (SBV) and epizootic haemorrhagic disease virus (EHDV). Among the *Culicoides* species, *Culicoides imicola* is the most extensively distributed across the world [2], and rapid changes to its distribution can be driven by environmental and demographic factors [3]. This is concerning because changes in distributions can lead to disease introductions in new geographical areas. For example, bluetongue was historically considered enzootic in tropical regions of the world. However, since 1998 the northward distribution of *C. imicola*, coupled with the presence of competent vectors *Culicoides obsoletus* and *C. pulicaris*, have driven the spread of bluetongue across southern European countries [4, 5]. It can now be found in all continents of the world, apart from Antarctica [6]. In comparison, in the last 80 years, AHS has been identified in southern Europe [7], the Far and Middle East [8], North Africa [9], Southeast Asia [10] and sub-Saharan Africa [11, 12]. Many of these outbreaks were more contained than BT outbreaks; however, due to the similarities in transmission dynamics, there is concern that AHS has the potential to mount widespread and devastating outbreaks in Europe, similar to those experienced in the 2006–2009 BT outbreak [11]. Moreover, concerningly, BT mortality typically ranges between 2 and 30% [13], whereas AHS mortality can range between 50 and 95% [14].

Understanding the distribution, as well as the drivers, of *Culicoides* can, therefore, provide insight into one factor that influences disease risk. A considerable number of studies have explored different *Culicoides* species distributions, and distribution maps for *C. imicola* have been compiled for Portugal, Morocco, Italy, Spain, the Mediterranean basin, Senegal, South Africa and even globally [2, 3, 15–24]. The number of studies and diversity of methods applied to characterising *Culicoides* distributions underscores the importance of having up-to-date and data-driven maps.

Machine learning can be utilised to predict the likelihood of an outcome occurring based upon a set of predictor variables. Typically, for predicting vector distributions, predictor variables include environmental and climate data which can either be locally collected from trap-site instruments, local weather stations, or remote sensing data, such as satellite imagery. Many machine learning techniques are available to model the presence or absence of a species, including random forest (RF) [25], support vector machines (SVM) [26], generalised linear models (GLM) [27], artificial neural networks (ANN) [28], generalised additive models (GAM) [27], multivariate adaptive regression splines (MARS) [29], MaxEnt [30], extreme gradient boosting (XGBoost) [31] and convolutional neural networks (CNN) [28]. Ensemble modelling is also possible, where multiple models are developed using different machine learning techniques and combined to create a final ensemble model [3, 20]. Predicting abundance is significantly more difficult than predicting presence/absence due to a number of factors, such as dispersal capabilities, microenvironment suitability, fine-scale biotic interactions, and handling datasets which demonstrate large variations in abundance between nearby locations [19]. However, understanding abundance is important when it comes to modelling vector-borne disease outbreaks, as the number of vectors may influence disease spread. The requirement for abundance data for this type of modelling, therefore, limits the machine learning techniques available, and random forests (RF) have been proven to outperform other methods for this type of prediction [19].

South Africa is a highly competent region of the world for BT and AHS, which often circulate endemically at low levels but can also sporadically cause seasonal peaking outbreaks. The two most competent vectors in South Africa for both diseases are *Culicoides imicola* and *C. bolitinos*. To date, only two models have explored vector distributions in South Africa. The first study was undertaken by Baylis, Meiswinkel and Venter (1999) [15] and analysed abundances of only *C. imicola* with linear regression and correlation coefficients to compile and evaluate two models based on different types of predictors. The first model used locally collected climatic predictors only whilst the second also included satellite data. The former explained only 34% of the variance whereas the latter accounted for nearly 67% of the variance. Since the date of that research, over two decades of *Culicoides* abundance data have been actively collected in the Western Cape in South Africa, so the knowledge on *Culicoides* spp. distribution has significantly changed. At a later date, Eksteen and Breetzke (2011) [22] were the first to use machine learning to predict midge abundance in South Africa. Artificial neural networks (ANN) were developed to predict locations with a high abundance of *C. imicola* and *C. bolitinos* combined. The model showed 83% accuracy; however, distribution maps were not developed, and the resulting data were only available as geographical points [22]. Since then, an additional decade of *Culicoides* trap data has also been recorded.

In South Africa, *C. bolitinos* abundance maps alone using machine learning predictive techniques have never been compiled, and published *C. imicola* abundance maps [15, 22] are now outdated and developed with limited data. In addition, interpolation mapping, which is a more simplified form of distribution mapping without using predictor variables that naively smooths data between points, has been explored previously for both *C. imicola* and *C. bolitinos* [32], but again these are now outdated, since 20 years of additional data have now been collected. Maps are vitally important to be able to develop disease models to investigate control and prevention of vector-borne diseases, such as bluetongue and African horse sickness. They also improve knowledge of the main drivers of *C. imicola* and *C. bolitinos* abundance. In this article, we mapped vector abundance in the Western Cape of South Africa using a RF method. RF prediction is based on multiple decision trees which are each made up of nodes, where predictor data split and the algorithm learns how to move through the tree to reach a final answer. In this case, environmental, climate and livestock density data were used as predictors to reach a final answer of how many midges were in an area. The performance of the model was explored by using 80% of the data to train the model and then testing with the remaining 20% of the data to understand how well the model predicted the number of midges. By developing the RF models, this research therefore addressed three main aims. The first aim was to develop *C. imicola* and *C. bolitinos* abundance maps and investigate whether they can be successfully predicted in South Africa using a RF approach. The second aim was to understand how the RF approach performed compared to interpolation mapping. The final aim explored whether *C. imicola* and/or *C. bolitinos* abundance was directly correlated with historical outbreak location.

## Methods

This research was based on an extension of the methodology presented by Cuéllar et al. (2020) [19], which modelled *C. imicola* using random forest machine learning. The methodology was extended by applying it to another geographical area (South Africa rather than Europe) as well as other *Culicoides* species (*C. bolitinos* and a merged population of AHS/BT competent vectors). In addition, further predictive variables were identified which to our knowledge have never before been used in *Culicoides* models, which included lagged variables, which may have an influence on the breeding and activity of midges one month prior to their collection date and livestock density variables. Finally, this research added an additional aim to explore whether historic outbreak location and midge abundance were correlated.

### *Culicoides* dataset

**Fig. 1:**
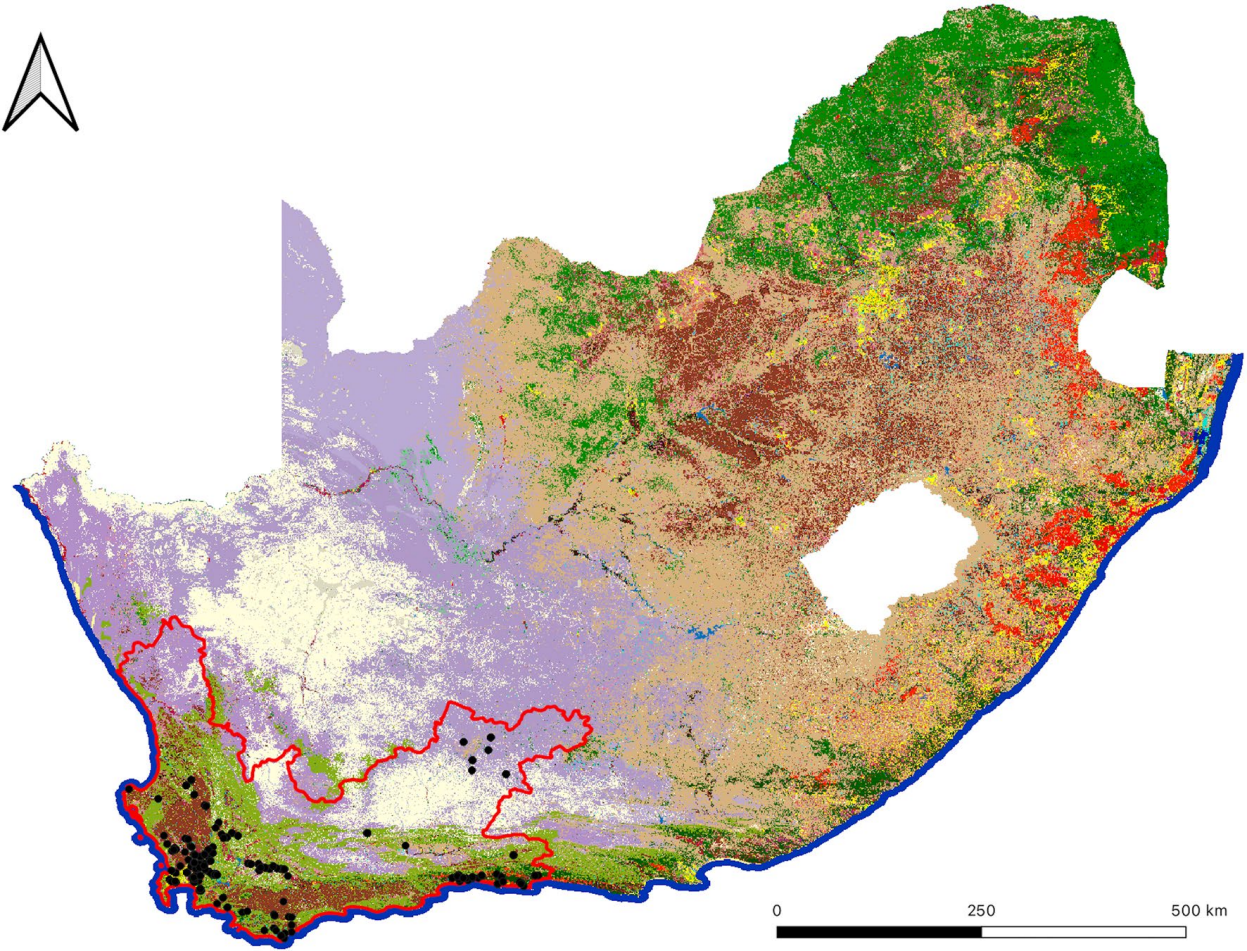
Map of the National Land Cover of South Africa (available from https://egis.environment.gov.za/sa_national_land_cover_datasets) with the Western Cape outlined in red and locations of midge trapping points used over 20 years indicated by black dots

**Fig. 2:**
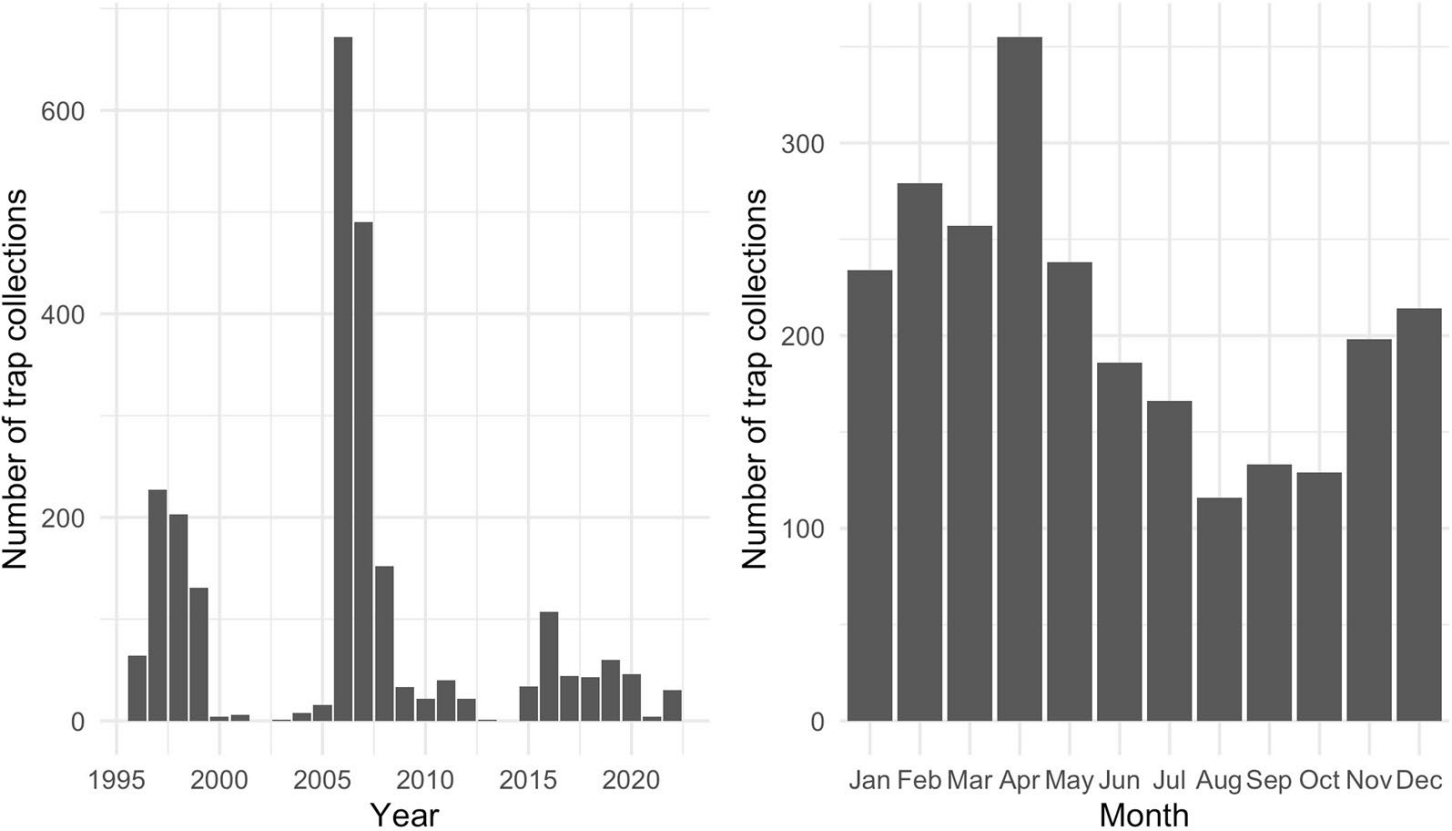
Number of trap collections over the study period in each year (left) and sum of all years for each month (right)

*Culicoides* trap data were provided by the Agricultural Research Council (ARC) in South Africa. *Culicoides* were collected between 1996 and 2022 nationally, which comprised 171 different sample locations (Fig. 1) in the Western Cape, South Africa, on 2505 trap collection dates (Fig. 2). Over the years represented in this study, the number of collections made per year varied depending on the study ARC was undertaking. In particular, there were two time periods which experienced more intensive sampling than other years. From 1996 to 1999 the data were collected as part of a country-wide EU project (EU Grant IC18-CT95-0010) and samples were collected weekly at selected farms. In addition, between 2006 and 2008 there was a study focussing on intensive sampling in the Stellenbosch area. However, for all studies, the methodology of the trapping always used Onderstepoort 220v UV light traps, where, after collection, the midges were then sorted from other insects and species identified. *Culicoides imicola* and *C. bolitinos* counts were used for this model, as these are the most competent vectors for BT and AHS in South Africa [32] with 80.55% and 2.87% of the national dataset comprising these species respectively. Five other species have been identified as competent field vectors of AHS or BT (but not both) in South Africa but cumulatively only account for 1.37%. In total, 6,183,986 specimens of *C. imicola* and 238,028 specimens of *C. bolitinos* were caught in the Western Cape, and over 44 million specimens of many species were caught nationally. Trap counts always represented one night’s collection, apart from the Kenilworth location, where it represented seven nights. For this reason, Kenilworth counts were divided by seven. The abundance data were then log-transformed to give a range between 0–5.5 for *C. imicola,* and 0–4.5 for *C. bolitinos*, or 0–5.5 when merged to create an AHS and BT competent vector dataset.

### Predictor data

Both locally sourced climatic data from the South African Weather Service and remotely sensed satellite data were used to develop the RF model together with estimates of cattle, sheep and horse distributions and land cover features. Predictors were compiled into a tabular format, which involved all data in raster format to be reprojected to match each other, and then values extracted at the GPS point coordinates of the trap sites on the closest matching date to the sampling date using R software (version 2023.06.1 + 524) and packages *terra* and *raster*. This difference was no more than 15 days.

The South African Weather Service provided tabular data for minimum and maximum daily temperature (Tmin and Tmax) and minimum and maximum wind, which was recalculated to give the mean daily wind (WND) by determining the value in the middle of the range between minimum and maximum. Average monthly precipitation values were also provided. All climate parameters were also recorded for 4 weeks prior to the sampling date as the lifespan length for *Culicoides* is largely temperature dependent but could range between 13.5 and 45 days for temperatures between 20 °C and 28 °C [33], with a mean length under laboratory conditions of 25.5 days for *C. imicola* [34]. *Culicoides bolitinos* has not been studied in a similar manner; however, the lifecycle length is likely to be similar, since most *Culicoides* species exhibit life cycles in this range [34]. However, adult phase *C. bolitinos* appear slightly hardier than *C. imicola* at lower temperatures [35], which may mean their life cycle length could be slightly longer in the winter. The mean distance to the nearest weather station was 178.6 km ± 140.9 km, median 115.1 km, and range from 13.6 km to 825.9 km. These data were used as predictors in the model but were not suitable for projection as they were point data. Standard interpolation using these data between weather stations could lose vital information because of the decreased density of the meteorological network in some geographical locations [36] but high-resolution WorldClim raster files are easily publicly available at 30 s (~ 1 km^2^) spatial resolution containing average data for each month spanning the previous 20 years, and these were used for projection [37]. WorldClim data are interpolated using weather station data and therefore are a closer match to the predictor data used to build the model than satellite data; a more complex method than standard interpolation is used to develop the maps and improve accuracy. This includes using thin-plate splines with covariates, such as distance to the coast, elevation, satellite-derived minimum and maximum land surface area and satellite-derived cloud cover [37]. The addition of satellite data combined with on-the-ground data in the development of these maps improved temperature prediction by up to 15%, and therefore this supported the use of WorldClim maps over standard interpolation methods.

Raster layers derived from Moderate Resolution Imaging Spectroradiometer (MODIS) datasets were downloaded, which contained normalized difference vegetation index (NDVI) and enhanced vegetation index (EVI) data between 2000 and 2022 [38]. To add to the data set of predictors, the raster files captured nearest to the date of sampling and 30 days prior (as images were captured every 15 days) were used to extract NDVI and EVI values at each point location. The same rasters were used for projection by developing 20-year mean NDVI and mean EVI raster layers for each month of the year. Similarly, the soil moisture raster layer was downloaded from the Copernicus Climate Data Store [39] and processed in the same manner.

Cattle and sheep population distribution raster layers and soil types were provided by the Western Cape Department of Agriculture [40]. Livestock distribution layers were calculated using interpolation mapping from census data between 2020 and 2023. Soil types were originally compiled by the National Department of Agriculture, Forestry and Fisheries, and categorised by the Environmental Potential Atlas of South Africa (ENPAT) soil description code, with broader categories for soil descriptions. In total, 1424 different ENPAT codes were recorded, of which 1038 only had one data point and therefore would not perform as a suitable predictor for training a RF model. Instead, these were sorted into 18 different categories based on the broader soil descriptions (Supplementary Material 1), which were input into the model.

The horse population distribution raster layer was provided by the South African Equine Health and Protocols Non-Profit Company (SAEHP) and compiled using population data collected during a 2002 census. This raster layer has previously been used for an exposure risk assessment for AHS introduction through the movement of horses into the Western Cape control zone [41] and to develop a disease transmission model of AHS in the same area [12].

The 2020 National land cover raster layer derived from 20 m Sentinel 2 data was downloaded from the National Department of Forestry, Fisheries and the Environment website [42]. The raster layer was comprised of 73 categories, grouped into nine description categories (Supplementary Material 1). Since the randomForest R-package is limited to 53 categories, and to optimise the prediction power of the RF, the nine description categories were used.

The Copernicus Climate Data Store was used to download aggregated monthly, quarterly or annual soil moisture, aridity, frost, water vapour, dry days and 19 bioclimatic variables (which underpin the 20-year average BIO variables from WorldClim) [43]. These raster layers had a lower resolution (0.5° × 0.5°, ~ 55 km^2^) than those from other sources and did not extend to the coast in the region of Mossel Bay and George, resulting in approximately 1% reduction in area of the Western Cape and loss of 53 out of 2505 midge captures. Therefore the “focal” function from the *Raster* package was used to calculate new values using a moving window for the neighbourhood of focal cells. The same weight was given to all regions of the focal window, and this allowed for a smoother transition from the values of one pixel to another. It also allowed for extension over the missing values at the coastline to the edge of the focal window.

Finally, a digital elevation model was downloaded from WorldClim, which was derived from Shuttle Radar Topography Mission (SRTM) elevation data at 30 s (~ 1 km^2^) spatial resolution [37].

All pairs of predictor variables were then explored for correlation using a correlation matrix, Kruskal-Wallis rank sum tests followed by Dunn’s tests, or Chi-squared tests, depending on whether the pairings were continuous or categorical, and one predictor from each highly correlated pair was removed to ensure the RF model was not built upon co-dependent predictors. A highly correlated continuous pairing was defined as a correlation of > 0.7 [44–47], > 50% of the categories of a variable being significantly associated with a continuous variable in a Dunn’s test or a *P*-value < 0.05 in a Chi-squared test.

All 53 predictor and projection variable sources are listed in Supplementary Material 1, with predictors that were removed being marked with an asterisk (Additional file 1: Table SM1).

### Random forest model

The randomForest package in R was used to predict the log_10_ transformed abundance of *C. imicola*, *C. bolitinos* and combined counts of competent midge species for AHS and BT, further referred to as “merged species”. Random forest (RF) is a machine learning method which can use classification or regression to come to an end result and predict an outcome. It is achieved through the development of decision trees, which make a series of decisions to reach a conclusion. This is then repeated many more times with other trees until the error rate for prediction is reduced as low as possible. The RF technique is well recognised for predicting vector abundance or presence [19, 48–50]. Several aspects contribute to its popularity including the ability to identify non-linear, complex relationships between predictors and the outcome variable as well as the ability to identify importance of predictors in the response and apply a ranking to them. It has also been identified as a method with very high sensitivity and specificity for modelling *Culidoides* distributions as well as excellent ROC, kappa and TSS scores [20].

The RF model used here was developed using 501 trees to ensure the minimum error had been reached and the m_try_ parameter tuned to six using the tuneRF function of the randomForest package, which is also the default for regression as it is the number of parameters divided by 3 rounded to the nearest integer [51]. For external validation of the model, the data for each midge species were randomly split into five folds, each containing 20% of the data. Four of the folds (80%) were used for training the model and the remaining one was used for testing. For analysis, the test data were plotted against the predicted values of the training data. The model was evaluated with residual plots, Q-Q plots (Additional file 1: Figure SM2) and importance graphs. This was repeated five times, using a method called k-fold cross validation, to examine how the model performs with different random subsets of the data. The model was then used to project predicted data into a raster layer. Midge trapping points were plotted over the projection and values extracted. The normalised root mean square error (nRMSE = RMSE/mean of predicted values) was then calculated, which is a measure of good fit. Normalisation of the root mean square error enabled direct comparison of projection rasters of different months, where lower values represented lower error and therefore better predictions.

### Model comparison to interpolation mapping

Another common method to explore spatial distribution of *Culicoides* spp. is through interpolation mapping. This method is relatively simple and does not require any predictors. The “interpolate” function of the *Raster* package was used to develop raster layers using inverse distance weighted interpolation. By this method, the further away an area was from a point, the less influence that point had on its value. The same k-fold cross-validation method was used, using five folds and five repeats, and nRMSE calculated for comparison to RF maps.

### Outbreak data comparison

Three months were chosen where there were multiple BT and AHS outbreaks recorded in the Western Cape [52]. These were April 2006, March 2011 and April 2014. These dates were chosen as there were multiple BT and AHS outbreaks in those months, and they were within a date range where rasters were available from those dates for projection of the model onto maps. A visual assessment of correlation between midge abundance and outbreaks was observed from the projected maps of both the month of the outbreak and midge abundance from the month prior to the outbreak. In addition, the *Culicoides* abundance was extracted from each data point and recorded along with the mean abundance for the Western Cape during the time of the outbreak and the month prior to the outbreak and Welch’s *t*-tests were performed to explore whether midge abundance in BT or AHS outbreak locations differed significantly from the mean of the study area.

## Results

### Random forest model performance

**Fig. 3:**
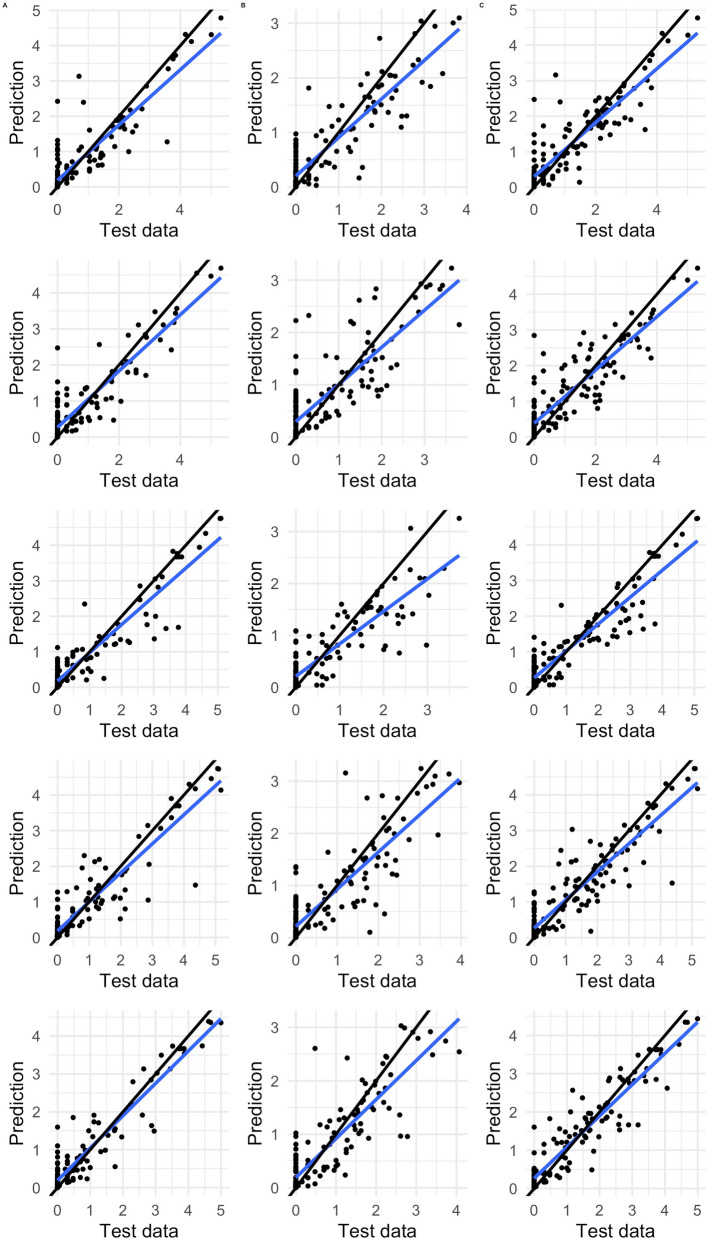
Test plots comparing the fivefold cross-validation results where test data (20% of the data) were plotted against predicted results (from the remaining 80% of the data) using the random forest model for each midge species. Each graph has different and random test and training data. Column **A** = *Culicoides imicola*, **B** = *C. bolitinos*, **C** = grouped competent AHS/BT vectors. The black line indicates a model where predicted data equal the test data. The blue line displays the line of best fit

The RF models compiled with 17 predictive layers, out of the 53 layers considered, explained the most variance in *C. imicola* and the merged species datasets (Table 1), with final compiled results accounting for 75.02% and 74.01% variance explained, respectively. The *C. bolitinos* model had slightly less variance explained with 61.6%. Predicted points using the model and 80% of the data were plotted against the remaining 20% of the data to explore how well the model fit the data (Fig. 3). This was repeated five times, using a k-fold cross-validation technique.

**Table 1:** Sources of prediction and projection variables

Source	Abbreviation	Prediction layer	Projection layer
South African Weather Service	Tmin	Minimum daily temperature (Celsius)	WorldClim monthly 20 year average Tmin
	Tmax	Maximum daily temperature (Celsius)	WorldClim monthly 20 year average Tmax
	Tmin_4w	Minimum daily temperature (4 weeks prior) (Celsius)	WorldClim monthly 20 year average Tmin (month prior)
	Tmax_4w	Maximum daily temperature (4 weeks prior) (Celsius)	WorldClim monthly 20 year average Tmax (month prior)
	WND	Average daily wind (m/s)	WorldClim monthly 20 year average wind
	WND_4w	Average daily wind (4 weeks prior) (m/s)	WorldClim monthly 20 year average wind (month prior)
	PRCP	Average monthly precipitation (mm)	WorldClim monthly 20 year average precipitation
	PRCP_1m	Average monthly precipitation (1 month prior) (mm)	WorldClim monthly 20 year average precipitation (month prior)
Copernicus CDS	SM	Volumetric soil moisture (m^3^ water per m^3^ soil)	Copernicus monthly soil moisture 20-year aggregated mean
	SM_annual	Volume of water in the top 7 cm of soil. Annually averaged	Copernicus annual soil moisture 20-year aggregated mean
	AR_dry	Monthly evaporation divided by monthly mean precipitation (m s^−1^). Average of the driest quarter	Copernicus aridity in the driest quarter 20-year aggregated mean
	FST	Number of days per year with temperature < 0 °C	Copernicus annual frost days sum 20-year aggregated mean
	WV	Daily water vapour pressure, aggregated monthly	Copernicus monthly water vapour 20-year aggregated mean
	BIO8	Mean temperature of the wettest quarter	Copernicus BIO8 20-year aggregated mean
WCDoA	CTL	Cattle distribution (2020–2023 average)	Same as prediction layer
	SHP	Sheep distribution (2020–2023 average)	Same as prediction layer
WorldClim	DEM	Digital elevation model	Same as prediction layer

Monthly subsets of the data were then used in the model. The nRMSE was calculated to compare each month’s predictive performance, using the same cross-validation method, and the mean nRMSE compared to other months and species. Lower nRMSE values indicated better performance of the model than higher. Overall, all three species maps performed well, with *C. imicola* maps achieving the lowest ranges of nRMSE results (0.62–1.22) compared to *C. bolitinos* (0.77–1.45) and merged species (0.71–1.59). All species maps had lower nRMSE values in the summer compared to the winter (Table 2).

**Table 2:** Table showing all nRMSE results to compare RF and interpolated predictive abilities

	*Culicoides imicola*		*Culicoides bolitinos*		*Merged*	
	RF	Interpolation	RF	Interpolation	RF	Interpolation
January	0.63^	0.67	0.86^	1.02	0.72	0.59^
February	0.62	0.52^	0.77^	0.93	0.71	0.45^
March	0.64	0.50^	0.82^	1.07	0.75	0.46^
April	0.68^	0.68^	0.86^	1.24	0.84	0.64^
May	0.80^	0.93	0.95^	1.48	0.95	0.92^
June	1.02	0.95^	1.11^	1.18	1.27	0.90^
July	1.22	1.13^	1.45^	1.48	1.59	1.08^
August	1.21^	2.53	1.05^	2.51	1.46^	2.45
September	1.12^	3.20	1.13^	1.97	1.50^	2.24
October	0.75^	1.08	0.92^	1.09	0.98	0.88^
November	0.75^	0.99	0.80^	0.87	1.09	0.71^
December	0.71^	1.11	0.91^	1.04	0.85	0.83^

^ indicates which map performed better for the species being modelled

**Fig. 4:**
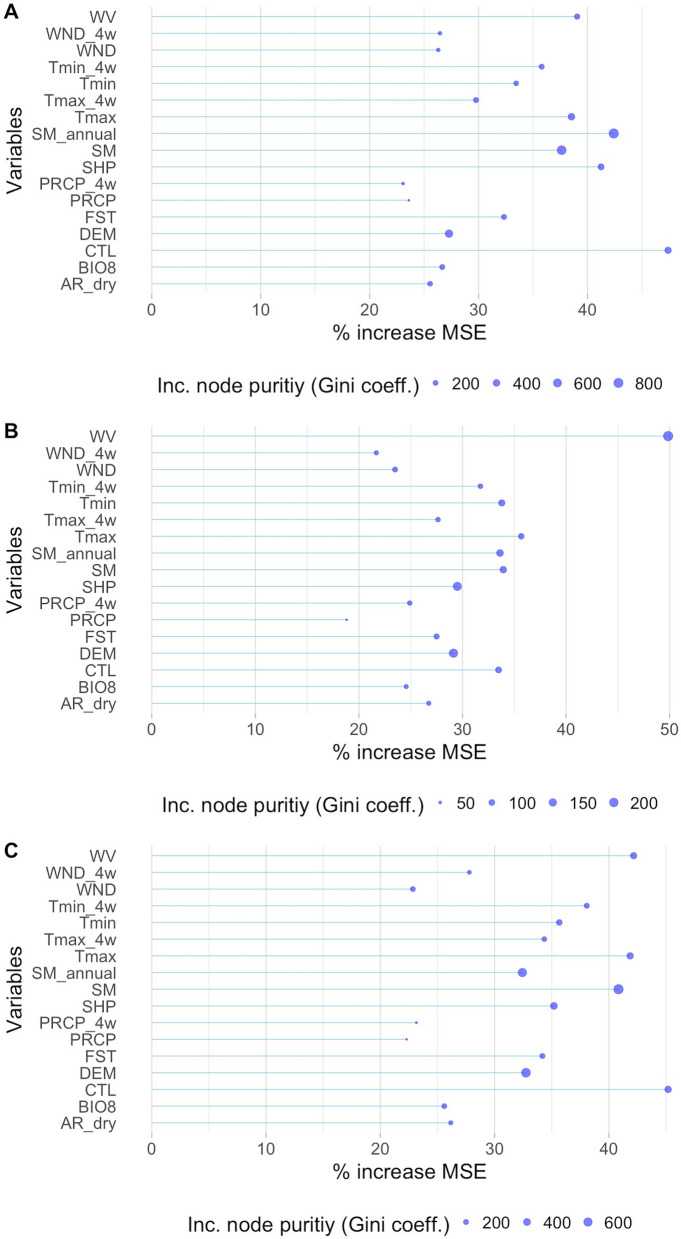
Importance graphs demonstrating which are the most important predictors for each midge species, random forest model. % increase in mean square error (MSE) is a measure of how much removing a variable decreases accuracy, and the Gini coefficient is a measure of how important a variable is when coming to choose it at a node in the random forest tree. **A** = *Culicoides imicola*, **B** = *C. bolitinos*, **C** = merged competent AHS/BT vectors

The two most influential predictor variables of importance for *C. imicola* were cattle density followed by annual soil moisture. This was similar to the merged model, however water vapour pressure and maximum daily temperature were also identified as important. Cattle density, soil moist and maximum temperature were also important predictor variables for *C. bolitinos*; however, water vapour pressure was the most influential (Fig. 4). Overall, in all three models, there were no predictors which contributed to a < 18% increase in mean square error if removed from the model.

**Fig. 5:**
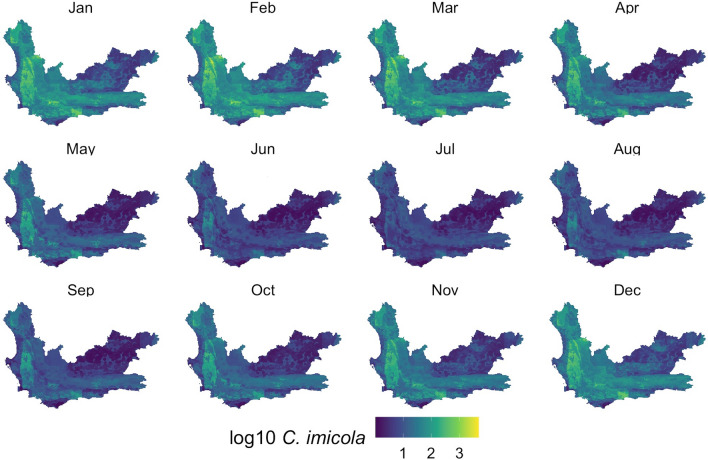
*Culicoides imicola* prediction in each month of the year. *Culicoides imicola* was predicted to be more abundant in the western and southern areas of the Western Cape, with the northeast being relatively low in abundance. The cooler months (May to September) demonstrated lower abundance

**Fig. 6:**
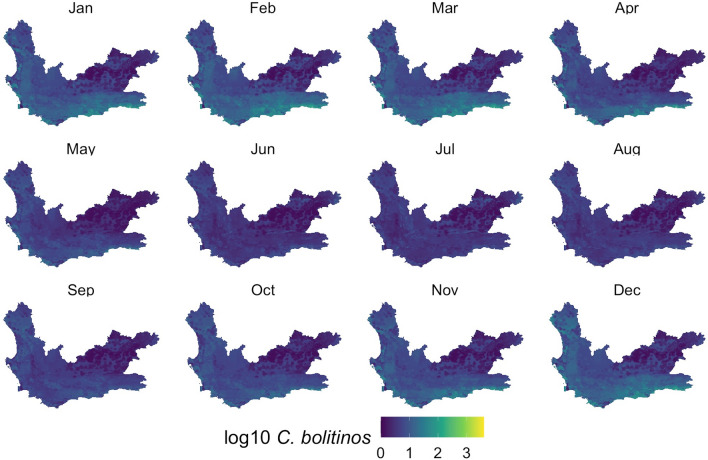
*Culicoides bolitinos* prediction in each month of the year. *Culicoides bolitinos* was predicted to be more abundant in the southern areas of the Western Cape, with the north being comparatively low in abundance. However, overall, the Western Cape displayed low abundance for *C. bolitinos*. The cooler months (May to September) demonstrated lower abundance

**Fig. 7:**
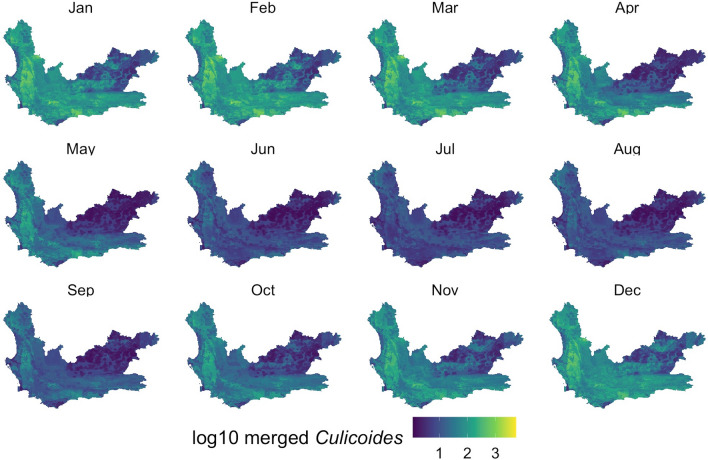
Merged competent vector prediction in each month of the year. Spatially, the merged competent vector population demonstrated a similar abundance pattern to *Culicoides imicola*

With the models developed, monthly *Culidoides* maps were then generated using monthly subsets of the predictor data (Figs. 5, 6, 7). Overall, all midge populations in the winter months of June, July and August had very low abundance, whereas from November through April, higher numbers were seen in the *C. imicola* and merged species populations. *Culicoides bolitinos* overall had a much lower abundance than *C. imicola*, as well as a shorter peak season from January to March. On a spatial scale, *C. imicola* appeared to be more abundant in the west and south of the Western Cape, whereas *C. bolitinos* favoured the south only.

### Comparison of interpolation and random forest models

Monthly interpolation maps were also generated for each month of the year using the midge capture data aggregated by month (Additional file 1: Figures SM3a–c). nRMSE was calculated using the k-fold cross-validation method by holding out 20% of the data and determining the ability of the interpolation map to predict this data, and this was used to compare each month’s predictive performance to the RF predictions. Compared to the interpolation method, RF achieved much smaller ranges of nRMSE throughout the year, indicating it was more consistent across all the seasons compared to interpolation mapping (Table 2). In particular, the later winter months of August and September received very high nRMSE scores for interpolation mapping compared to RF predictions. When *C. imicola* and *C. bolitinos* were mapped individually, they performed better than interpolation maps; however when merged, the interpolation maps on average performed slightly better throughout most of the year. Nevertheless, when analysing the whole year and not seasonal trends, t-tests did not indicate any statistical differences for *C. imicola* (*P* = 0.19) or merged species (*P* = 0.83), but there was a statistical difference for *C. bolitinos* (*P* = 0.03).

Visually assessing the RF maps (Figs. 5, 6 and 7) in relation to to the interpolation maps (Additional file 1: Figures SM3a-c), the RF model achieved a comparatively smoothed out prediction. The RF maps did not display any focal points of very high counts, as seen in the interpolation maps and capture data. These very high peaks, particularly in the *C. imicola* data, only occurred in the warmer months, and therefore the interpolation maps mostly performed better for these months as they could account for the peaks, whereas the RF maps predicted better for the colder months which had a lower range of abundance.

### Outbreak locations

**Fig. 8:**
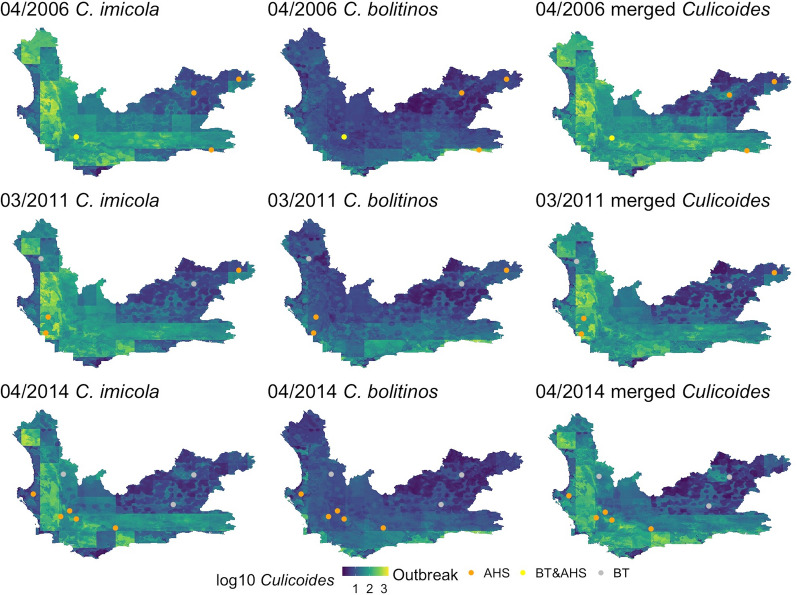
Point locations of AHS and BT outbreaks in April 2006 (first row), March 2011 (second row) and April 2014 (third row) according to the DAFF disease database [52] plotted over the *Culicoides imicola* (first column), *C. bolitinos* (second column) and merged *Culicoides* species (third column) prediction maps. The months were chosen as they fell within the study period and were the three months with the most combined AHS and BT outbreaks

**Fig. 9:**
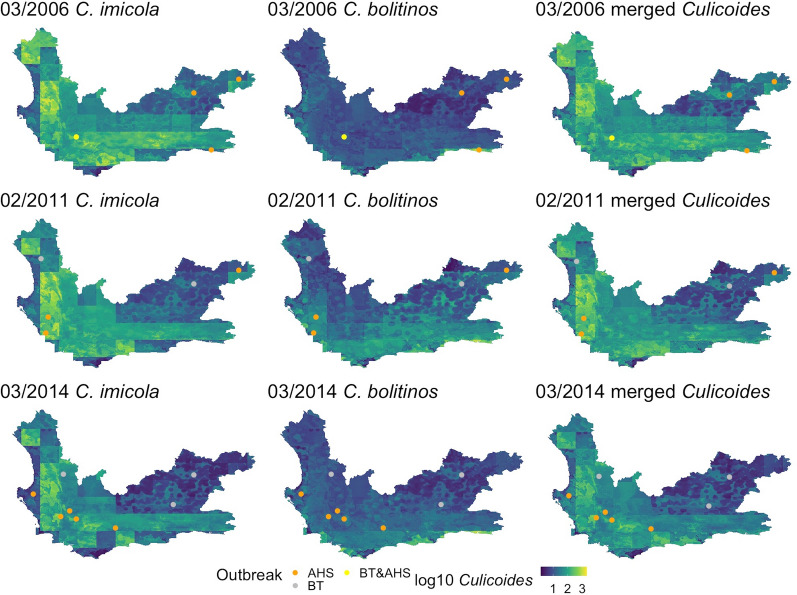
Point locations of AHS and BT outbreaks in April 2006 (first row), March 2011 (second row) and April 2014 (third row) according to the DAFF disease database [52] plotted over the *Culicoides imicola* (first column), *C. bolitinos* (second column) and merged *Culicoides* species (third column) prediction maps for the month prior to the outbreaks to explore whether there was a lagged occurrence of outbreaks after high midge counts

Point locations of BT and AHS outbreaks from three months within the study period that experienced multiple outbreaks were plotted over maps developed from predictor data specific to each date (Fig. 8) and the month prior (Fig. 9).

The figures did not display any obvious visual correlation between outbreak locations and predicted midge densities in that month. The t-tests comparing mean midge counts at all outbreak locations and mean midge counts overall for the map on each date showed no significant difference. Furthermore, there were no significant differences between mean midge counts for AHS locations of all the dates and the months prior in each species compared to the mean of all the midge counts of all the years, or BT, except for *C. bolitinos*, which indicated that BT locations had a significantly lower mean midge count during the month of the outbreak and month prior (0.68 and 0.72 respectively) compared to the average mean of the maps (0.87 and 0.99 respectively) (*P* = 0.027 and *P* = 0.021 respectively). However, there were very few points in this analysis (15 AHS and seven BT over all three months) and therefore this needs to be repeated with a larger dataset exploring more months.

## Discussion

In this study, the two main competent vectors of bluetongue and African horse sickness in South Africa were modelled using a random forest approach and compared to interpolation mapping. The need for abundance modelling is great, as these maps directly inform disease transmission models for policy makers to enable them to make the most appropriate decisions. The model incorporated a large dataset containing 26 years of consistently collected Culicoides trap counts in the Western Cape region of South Africa and therefore provides an opportunity to gain greater understanding of the ecology of these vectors. The *Culicoides* maps developed by this study overall had a good predictive power (Table 2) and explained a large amount of variance compared to previous studies [15, 19]. The predictive power was better in the summer months and reduced in the winter months for all species populations, which might be attributable to less sampling in these months or the fact that *Culicoides* also seek inside refuge, resulting in less correlation between ambient predictor variables and abundance than expected [53].

Spatial uncertainty can also be introduced if the raster layers of the parameters which contributed more importance did not have a fine enough resolution to capture some of the more locally concentrated details of the predictor variable. These farm-level variables are likely to contribute to the development of microhabitats which do not follow the trends of the neighbouring areas. They may include differences in farming practices from one farm to another, such as insecticide use, or infrastructure contributing to shelter or conversely creation of wind tunnels, or standing water from poor drainage or leaking irrigation pipes, as well as cleanliness of farmyards and focal areas of dung management. On a residential level, the temperate mild climate in the southern and central Western Cape provides the ideal climate for residents to develop lush gardens with focal areas of compost, contributing further to microhabitat formation. These microhabitats are essential to the life cycle of midges. For example, *C. imicola* is known to breed in moist organically enriched soils such as those created by leaking irrigation points [54] and *C. bolitinos* breeds in cattle, wildebeest and buffalo dung [55]. In residential areas, compost usually consists of a mixture of dung and vegetation. *Culicoides* species utilise a wide variety of breeding media from soil, leaflitter (in gutters or rock or tree cavities), dung, rotting fruit, and areas containing both soil and dung. Even though in residential areas there are probably fewer livestock hosts than in farm areas, *Culicoides* species are opportunistic feeders and will feed on large mammals, small mammals, birds [56, 57] and even humans [57]. In most gardens in South Africa, at night there are birds roosting in the trees and active small rodents, which helps sustain small *Culicoides* populations.

While some of the satellite data used in this analysis had a lower resolution than other predictor variables, the presence of factors leading to microhabitats can only be established on the ground level and not by satellite imagery and therefore likely would not have been captured even at a higher resolution. Overall, *C. imicola* models performed slightly better than *C. bolitinos* in the random forest model, suggesting that additional predictive variables or consideration of microhabitats may be required to achieve a more accurate prediction for *C. bolitinos*, and further fieldwork could contribute to collecting these data. Microhabitats can also contribute to lack of reliability of interpolation maps, as these maps are developed entirely on trap counts alone. If a high trap count was recorded, but because of a localised microhabitat such as a pile of dung close to the trap, this could result in the map incorrectly displaying high numbers of midges in the surrounding areas. Therefore, this also supports further fieldwork to collect data on observations of local surroundings to improve the reliability.

Aside from factors included in microhabitat formation, the main drivers for *C. imicola* distribution in previous European studies have included annual mean temperature, where temperatures have remained high throughout the year [24, 58], or land surface temperature [19] or precipitation, since *C. imicola* occurrence is more common when the annual rainfall is < 700 mm [24, 59]. In this study, while daily maximum temperature featured consistently as an important predictor, the presence of cattle was more important in the accuracy of the model for both *C. imicola* and *C. bolitinos*. Water vapour pressure was also an important predictor variable, particularly for *C. bolitinos*, which may be due to humidity playing a role in flight initiation [60]. *Culicoides bolitinos* is also known to breed in cattle, wildebeest and buffalo dung [55], which at higher temperatures and drier climates can dry out and break up. This is likely to have contributed to the lower abundance, particularly in the northeastern areas during the summer breeding season, where extremely high temperatures can be experienced.

This study highlighted the importance of considering environmental and climatic predictor variables when predicting midge abundance. When visually comparing the interpolation maps to the RF modelled maps using predictor variables, many marked differences were evident. Both sets of maps predicted that there were more *C. imicola* midges to the west than east and, across all species, more midges in the warmer than colder months. However, the RF maps were able to predict midge distributions with much greater detail and omitted any large hotspots or dead zones in the distribution of count data. This is a common result of RF predictions, where typically they underestimate higher values and overestimate lower values [19, 61]. Interpolation mapping, however, had many focal points, which differed greatly from the surrounding areas. Even though the nRMSE results suggested that interpolation mapping performed better in some warmer months for some species, there were still large areas of the study area where there were no points to calculate the nRMSE, such as the northwest and central areas, for the Western Cape (Fig. 1). Some of the features of these areas preclude the ability to place trapping sites, such as a mountain range running through the region. However, to further validate these maps, a more homogeneously distributed set of point data is essential. This, combined with a high density of trap sites, would contribute to more reliable interpolation maps, as the further away from a point an area is on an interpolation map, the less influence the point has on that value, and therefore it gradually moves towards the mean value. Since interpolation mapping does not consider environmental conditions, any results that indicate interpolation mapping predicts better than RF models must be interpreted with great caution because of the lack of homogeneously spaced point data.

This study has entirely focussed on abundance mapping; however, previous modelling attempts for *Culicoides* have more commonly focussed on modelling presence or absence of the midges and has shown some favourable results [3, 17, 62]. However, vector abundance is a vital datum for informing disease transmission models. There have been fewer attempts at modelling *Culicoides* abundance, most of which have not been able to produce similarly reliable results [19]. This is not surprising since the local abundance is not just reliant on habitat suitability but also on biotic interactions, farm management, microhabitat formation and stochastic effects [63]. This work could be further extended with a meta-analysis to compare how predictor variables affect abundance compared to presence or absence and whether they differ in any way.

To date, there has only been one other paper using machine learning methods to predict the occurrence of *Culicoides* vectors in South Africa [22], in which the study produced points of high abundance of merged *C. imicola* and *C. bolitinos* for the months of January to May. In comparison, the RF maps developed in this study for merged species largely agreed with the placement of the points identified by the other study, indicating that ecological niche modelling could be reproduced with other machine learning methods. Nevertheless, this study chose RF because very few machine learning methods can predict abundance data, and of those which can, RF has proven to be the most robust and accurate method [48, 64]. For this reason, previous methods using ANN were not replicated.

Similar to this study, Eksteen and Breetzke (2011) [22] also mapped the locations of the outbreaks of AHS over the ANN results. They found in areas where there were more trap counts for development of the model, the areas which experienced outbreaks were correlated with areas with predicted high abundance of midges. However, in areas with fewer midge trapping sites, and poorer data coverage, the model did not predict high numbers of midges in those areas. While the distribution of data was concluded to be a major contributing factor to the ability to predict an outbreak, local vaccination coverage against AHS and local livestock and equine management practices, such as the use of fly repellents and “Sweet Itch” rugs, can also alter the correlation between areas experiencing disease outbreaks and areas of high abundance. In our study, the prediction maps for each month and the month prior did not reliably act as an indicator of disease presence, but this analysis was limited by the number of outbreaks and exploration of all outbreaks during the study period would be beneficial to confirm this conclusion. Further work to predict more months leading up to an outbreak and exploring correlations with locations of outbreaks could be undertaken to determine whether the midge population density prior to an outbreak is more influential than the midge population density at the time of the outbreak as in this study only the month prior was explored. In agreement with Eksteen and Breetzke (2011) [22] and another study by Liebenberg *et al.* (2015) exploring the influence of climatic variables on the distribution of AHS outbreaks [65], local management practices and anthropogenic factors probably play a role, and therefore midge abundance alone is unlikely to be the sole factor influencing outbreaks. Another study exploring prediction of BT outbreaks using machine learning techniques identified that midge abundance needed to be combined with environmental variables as well as host variables to successfully predict outbreaks [66]. It also identified that while midge abundance was a main predictor for BTV-1, temperature and goat density were identified as more important predictors for BTV-4. Similar work has also been undertaken to explore AHS suitability across the world between 2020 and 2060, and the main predictors were found to be solar radiation, maximum temperature and precipitation variables rather than midge abundance [67]. Therefore, to reliably predict disease outbreaks using machine learning, midge abundance actually needs to be used as a predictor variable and combined with other predictor variables such as those used in the midge models as well as vaccination coverage and farm management practices. For example, in this analysis, local herd immunity through naturally acquired immunity or vaccination may have also contributed to lack of outbreak detection. Annual AHS vaccination is compulsory in many regions of the Western Cape and coverage is approximated to be > 90%, except for the AHS surveillance and free zones close to Cape Town and surrounding areas where vaccination can only occur with permission and therefore coverage is approximately 50% [12]. Bluetongue vaccination is not compulsory in South Africa; however, most farmers vaccinate at least their maiden ewes each year [personal communication, J. Kotze, State Veterinarian, 06/02/2023]. In South Africa cattle are not vaccinated and often develop sub-clinical infections which serve as a silent reservoir. This results in outbreak occurrence without detection which adds complexity to this analysis. However, many older cattle have acquired natural immunity, which may result in a lack of outbreaks in areas with high midge densities where outbreaks would be expected.

Alternatively, instead of using machine learning to understand outbreak risk with predictor variables that include midge abundance, climate variables, vaccination and farm management practices, midge abundance data can be used to feed mechanistic transmission models to understand disease outbreak risk better. This allows for exploration of how natural immunity and vaccination protocols are likely to affect outbreak locations.

In conclusion, the first aim of this study was to investigate whether *C. imicola* and *C. bolitinos* can be successfully predicted in South Africa using a RF approach. The models developed in this study were able to distinguish between midge distributions in different areas or months by using environmental and climatic predictors, with *C. imicola* performing slightly better than *C. bolitinos*. Compared to previous modelling studies, the models in this study performed well; however, further work to identify factors that contribute to their distributions that are not included in these models still needs to be undertaken. This could be undertaken at the farm-level, exploring whether on-farm management and infrastructure contribute to microhabitat development on a finer scale than what is evident on satellite data. The second aim was to explore how RF mapping compared to interpolation mapping. Throughout the year, there were more months when predicting individual species performed better in the RF maps, but interpolation maps performed slightly better when species were merged, probably because of the diluting of variables of importance for each individual species when combining them. The third aim was to explore whether there was a correlation between historical disease outbreaks and midge abundance. No reliable correlation was determined; however, several reasons contributed to this result. First, the dataset for this part of the study was limited, and significantly more dates must be analysed to fully conclude whether there is a correlation between midge abundance and outbreak location. Second, other variables contribute towards the likelihood of outbreaks occurring in certain locations, such as vaccination coverage, natural immunity and surveillance to detect sub-clinical infections. Therefore, this requires a much more complex approach to analysis to understand how midge abundance may influence outbreak location.

Overall, this work on *Culicoides* abundance in South Africa was particularly important because it is a highly competent area of the world for arboviruses such as AHS and BT. With more in-depth knowledge of *Culicoides* distributions, these diseases can be more accurately modelled and studied to produce results which are highly valuable for informing policymakers that are working towards the control and reduction of these diseases.

### Supplementary Information

Additional file 1.

## Data Availability

Most of the predictor variable data supporting the conclusions of this article are available from referenced locations included within the article. The predictor variables provided by the Western Cape Department of Agriculture can be requested from Dr Lesley Van Helden (lesley.vanhelden@westerncape.gov.za). Culicoides collection data are property of the Agricultural Research Council and can be obtained from Dr Karien Labuschagne (labuschagnek@arc.agric.za).

## Abbreviations

BT: Bluetongue
AHS: African horse sickness
RF: Random forest
MODIS: Moderate resolution imaging spectroradiometer
NDVI: Normalised difference vegetation index
EVI: Enhanced vegetation index
WCDoA: Western Cape Department of Agriculture
