# Supplementary material for: Modelling bluetongue and African horse sickness vector (Culicoides spp.) distribution in the Western Cape in South Africa using random forest machine learning
Source: Parasit Vectors. 2024 Aug 21;17:354. doi: 10.1186/s13071-024-06446-8 (PMC11340078; doi:10.1186/s13071-024-06446-8)
Supplement: Supplementary file 1 — Additional file 1. [file 13071_2024_6446_MOESM1_ESM.docx]

# Supplementary Material

SM1: Complete list of predictors analysed before inclusion in the model

Table SM1: Sources of prediction and projection variables. * Removed due to high correlation with another variable

| **Source** | **Abbreviation** | **Prediction layer** | **Projection layer** |
| --- | --- | --- | --- |
| South African Weather Service | Tmin | Minimum daily temperature (Celsius) | WorldClim monthly 20 year average Tmin |
|  | Tmax | Maximum daily temperature (Celsius) | WorldClim monthly 20 year average Tmax |
|  | Tmin_4w | Minimum daily temperature (4 weeks prior) (Celsius) | WorldClim monthly 20 year average Tmin (month prior) |
|  | Tmax_4w | Maximum daily temperature (4 weeks prior) (Celsius) | WorldClim monthly 20 year average Tmax (month prior) |
|  | WND | Average daily wind (m/s) | WorldClim monthly 20 year average wind |
|  | WND_4w | Average daily wind (4 weeks prior) (m/s) | WorldClim monthly 20 year average wind (month prior) |
|  | PRCP | Average monthly precipitation (mm) | WorldClim monthly 20 year average precipitation |
|  | PRCP_1m | Average monthly precipitation (1 month prior) (mm) | WorldClim monthly 20 year average precipitation (month prior) |
| MODIS | NDVI* | NDVI (closest date 15 days) | NA – removed |
|  | NDVI_1m* | NDVI (closest date 1 month prior) | NA – removed |
|  | EVI* | EVI (closest date 15 days) | NA – removed |
|  | EVI_1m* | EVI (closest date 1 month prior) | NA – removed |
| Copernicus CDS | SM | Soil moisture monthly average (% saturated) | Copernicus monthly soil moisture 20-year aggregated mean |
|  | SM_1m* | Soil moisture previous monthly average (% saturated) | NA – removed |
|  | SM_annual | Volume of water in the top 7cm of soil. Annually averaged. | Copernicus annual soil moisture 20-year aggregated mean |
|  | SM_dry* | Volume of water in the top 7cm of soil. Average of the driest quarter. | NA – removed |
|  | SM_wet* | Volume of water in the top 7cm of soil. Average of the wettest quarter. | NA – removed |
|  | SM_warm* | Volume of water in the top 7cm of soil. Average of the warmest quarter. | NA – removed |
|  | SM_cold* | Volume of water in the top 7cm of soil. Average of the coldest quarter. | NA – removed |
|  | AR_annual* | Monthly evaporation divided by monthly mean precipitation (m s^-1^). Annual average. | NA – removed |
|  | AR_dry | Monthly evaporation divided by monthly mean precipitation (m s^-1^). Average of the driest quarter. | Copernicus aridity in the driest quarter 20-year aggregated mean |
|  | AR_wet* | Monthly evaporation divided by monthly mean precipitation (m s^-1^). Average of the wettest quarter. | NA – removed |
|  | AR_warm* | Monthly evaporation divided by monthly mean precipitation (m s^-1^). Average of the warmest quarter. | NA – removed |
|  | AR_cold* | Monthly evaporation divided by monthly mean precipitation (m s^-1^). Average of the coldest quarter. | NA – removed |
|  | FST_month* | Number of days per month with temperature below 0 ^o^C | Copernicus monthly frost days 20-year aggregated mean |
|  | FST | Number of days per year with temperature below 0 ^o^C | Copernicus annual frost days sum 20-year aggregated mean |
|  | WV | Daily water vapour pressure, aggregated monthly | Copernicus monthly water vapour 20-year aggregated mean |
|  | DRY* | Number of days per year with <2mm rainfall | NA – removed |
|  | BIO1* | Yearly mean of the monthly mean temperature. | Copernicus BIO1 20-year aggregated mean |
|  | BIO2* | Monthly average of the maximum minus the minimum temperature | NA – removed |
|  | BIO3* | Monthly diurnal range divided by annual range * 100 | NA – removed |
|  | BIO4* | Standard deviation of the monthly average temperature * 100 | NA – removed |
|  | BIO5* | Monthly average of maximum temperature in month with the highest temperature | NA – removed |
|  | BIO6* | Monthly average of minimum temperature in the month with the lowest temperature | Copernicus BIO6 20-year aggregated mean |
|  | BIO7* | BIO5 minus BIO6 | NA – removed |
|  | BIO8 | Mean temperature of the wettest quarter | Copernicus BIO8 20-year aggregated mean |
|  | BIO9* | Mean temperature of the driest quarter | NA – removed |
|  | BIO10* | Mean temperature of the warmest quarter | NA – removed |
|  | BIO11* | Mean temperature of the coldest quarter | NA – removed |
|  | BIO12* | Annual mean precipitation | NA – removed |
|  | BIO13* | Maximum monthly precipitation. | NA – removed |
|  | BIO14* | Minimum monthly precipitation | NA – removed |
|  | BIO15* | Annual coefficient of variation of the monthly precipitation | NA – removed |
|  | BIO16* | Mean precipitation of the wettest quarter | NA – removed |
|  | BIO17* | Mean precipitation of the driest quarter | NA – removed |
|  | BIO18* | Mean precipitation of the warmest quarter | NA – removed |
|  | BIO19* | Mean precipitation of the coldest quarter | NA – removed |
| WCDoA | CTL | Cattle distribution (2020-2023 average) | Same as prediction layer |
|  | SHP | Sheep distribution (2020-2023 average) | Same as prediction layer |
|  | ENPAT* | Soil type (by description) | Same as prediction layer |
| SAEHP | HRS* | Horse distribution (2002 census) | NA – removed |
| DFFE | NLC* | National land cover (simplified categories) | NA – removed |
| WorldClim | DEM | Digital elevation model | Same as prediction layer |

Definitions: WCDoA = Western Cape Department of Agriculture, SAEHP = South African Equine Health and Protocols, DFFE = Department of Forestry, Fishery and the Environment.

### ENPAT soil description categories

Ab = Red-yellow apedal, freely drained soils; red, dystrophic and/or mesotrophic

Ac = Red-yellow apedal, freely drained soils; red and yellow, dystrophic and/or mesotrophic

Ae = Red-yellow apedal, freely drained soils; red, high base status, > 300 mm deep (no dunes)

Ag = Red-yellow apedal, freely drained soils, red, high base status, < 300 mm deep

Ba = Plinthic catena: dystrophic and/or mesotrophic; red soils widespread, upland duplex and margalitic soils rare

Bb = Plinthic catena: dystrophic and/or mesotrophic; red soils not widespread, upland duplex and margalitic soils rare

Ca = Plinthic catena: undifferentiated, upland duplex and/or margalitic soils common

Da = Prismacutanic and/or pedocutanic diagnostic horizons dominant, mainly red B horizons

Db = Prismacutanic and/or pedocutanic diagnostic horizons dominant, B horizons mainly not red

Fa = Glenrosa and/or Mispah forms (other soils may occur), lime rare or absent in the entire landscape

Fb = Glenrosa and/or Mispah forms (other soils may occur), lime rare or absent in upland soils but generally present in low-lying soils

Fc = Glenrosa and/or Mispah forms (other soils may occur), lime generally present in the entire landscape

Ga = Soils with a diagnostic ferrihumic horizon, predominantly deep (Lamotte form)

Gb = Soils with a diagnostic ferrihumic horizon, predominantly shallow (Houwhoek form)

Hb = Grey regic sands and other soils

Ia = Miscellaneous land classes, undifferentiated deep deposits

Ib = Miscellaneous land classes, rocky areas with miscellaneous soils

Ic = Miscellaneous land classes, very rocky with little or no soils

### National Land Cover (SALLC_1) description categories and SALLC_2 categories which have been condensed

Barren (Consolidated, Unconsolidated)

Buildings (Residential, Village, Smallholdings, Urban Vegetation, Commercial, Industrial, Transport)

Cultivated (Permanent Crops, Temporary Crops, Fallow Lands and Old Fields)

Forest (Natural Wooded Land, Planted Forest)

Grassland (Natural Grassland)

Quarry (Surface Infrastructure, Extraction Sites, Waste and Resource Dumps)

Shrubland (Shrubs, Karoo and Fynbos Shrubland)

Waterbody (Natural Waterbodies, Artificial Waterbodies)

Wetland (Herbaceous Wetlands, Woody Wetlands)

## SM2: Q-Q plots


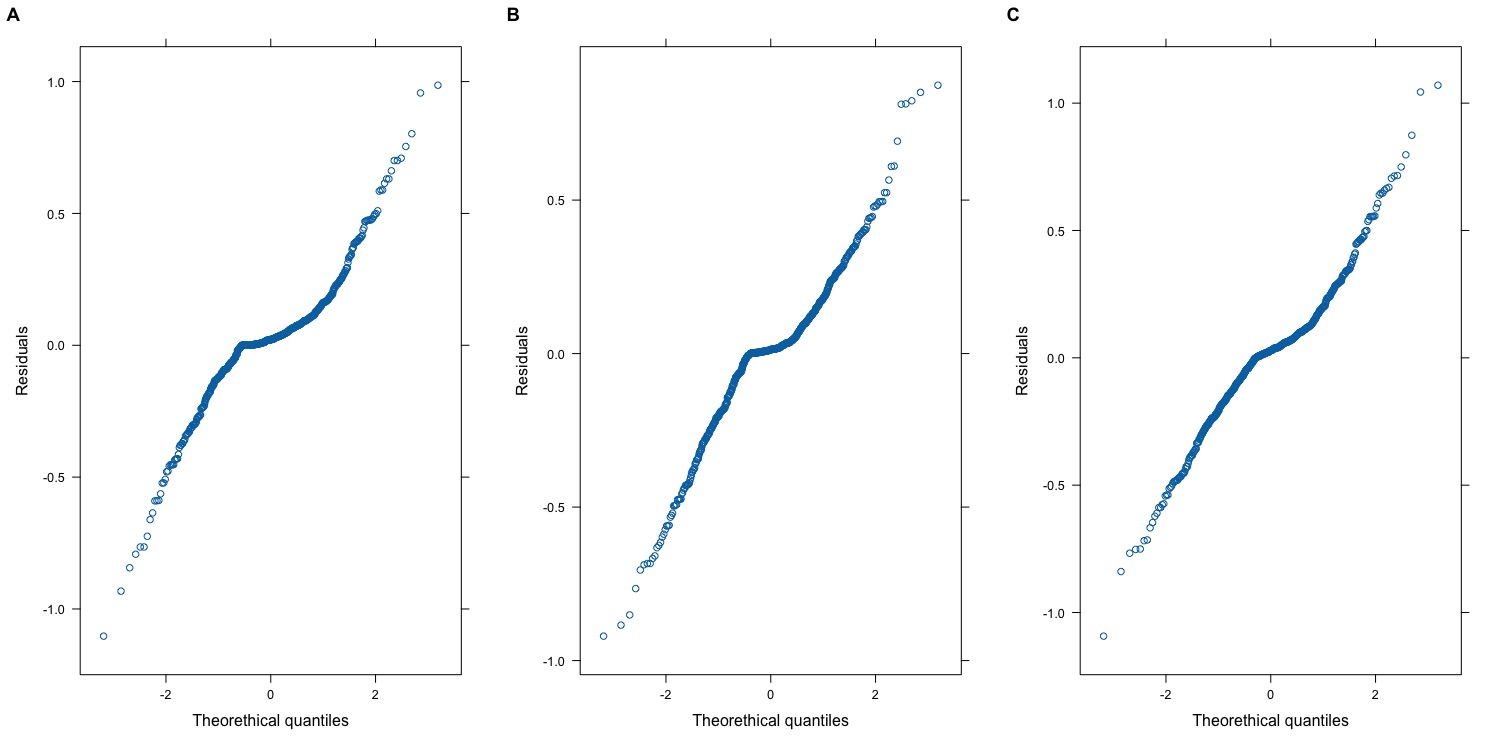


Figure SM2 Q-Q plots analysing the distribution of the residuals of each model. A = C. imicola, B = C. bolitinos, C = Grouped competent AHS/BT vector

## SM3: Interpolation maps


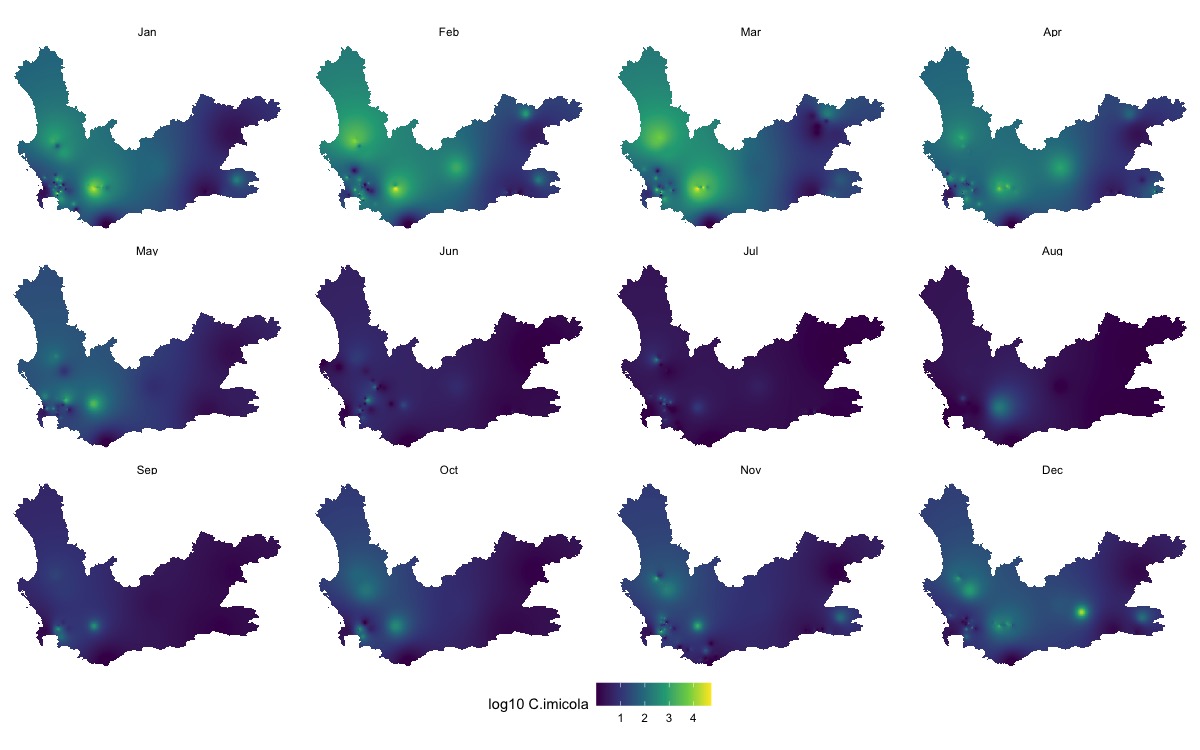


Figure SM3a C. imicola interpolation map


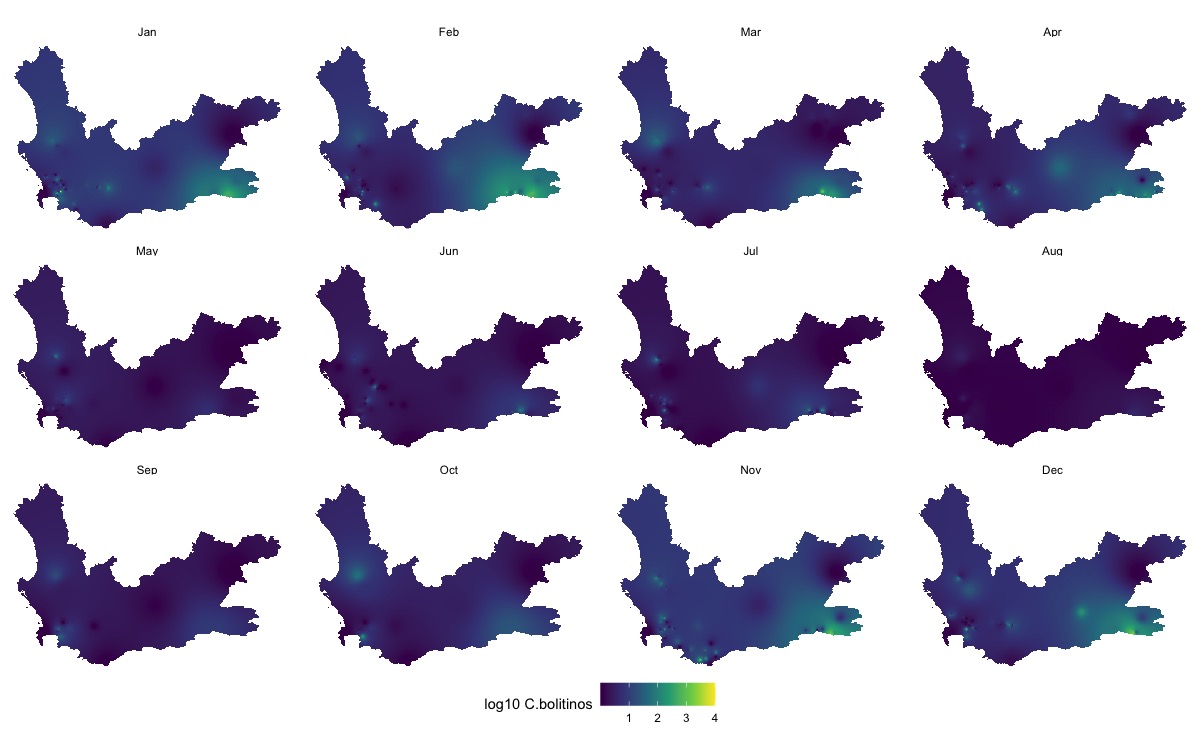


Figure SM3b C. bolitinos interpolation map


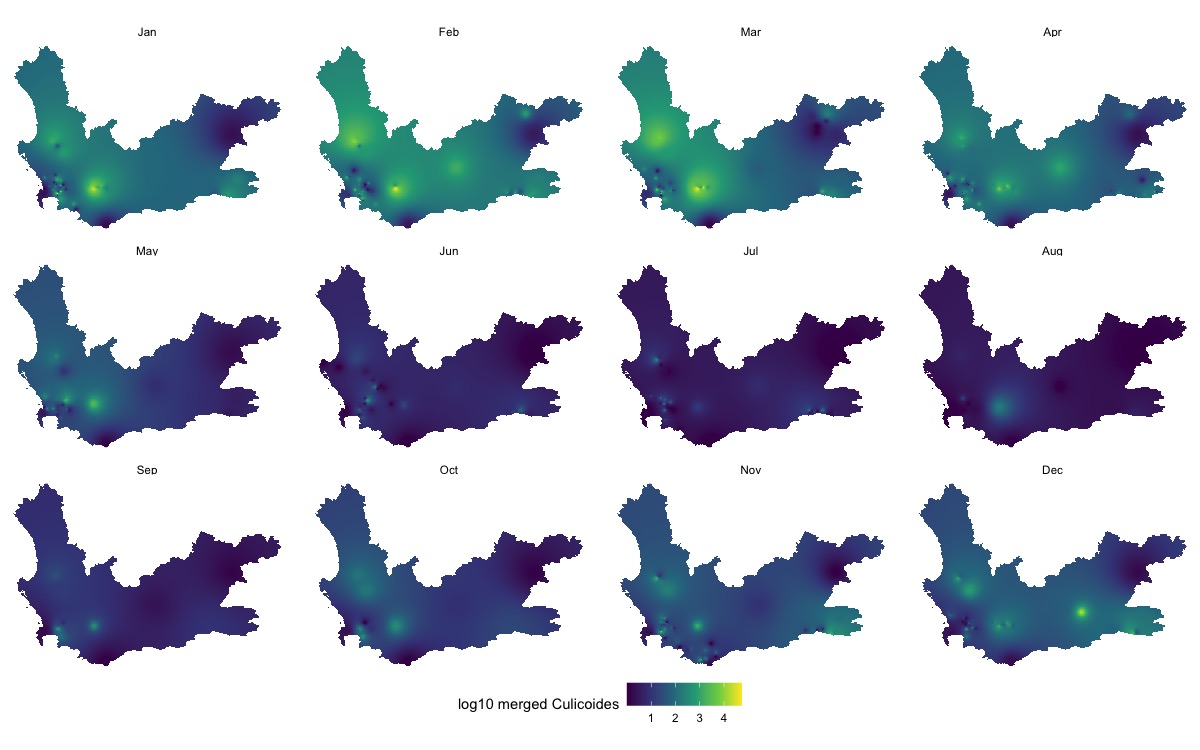


Figure SM3c Grouped competent vectors interpolation map
